# Genetic and epigenetic characterization of hypodiploid acute lymphoblastic leukemia

**DOI:** 10.18632/oncotarget.6000

**Published:** 2015-10-30

**Authors:** Setareh Safavi, Linda Olsson, Andrea Biloglav, Srinivas Veerla, Molly Blendberg, Johnbosco Tayebwa, Mikael Behrendtz, Anders Castor, Markus Hansson, Bertil Johansson, Kajsa Paulsson

**Affiliations:** ^1^ Division of Clinical Genetics, Department of Laboratory Medicine, Lund University, Lund, Sweden; ^2^ Department of Clinical Genetics, University and Regional Laboratories, Region Skåne, Lund, Sweden; ^3^ Division of Oncology and Pathology, Department of Clinical Sciences Lund, Lund University, Lund, Sweden; ^4^ Department of Pediatrics, Linköping University Hospital, Linköping, Sweden; ^5^ Department of Pediatrics, Skåne University Hospital, Lund University, Lund, Sweden; ^6^ Division of Hematology, Skåne University Hospital, Lund University, Lund, Sweden

**Keywords:** acute lymphoblastic leukemia, hypodiploidy, next generation sequencing, chromosomal instability

## Abstract

**Purpose:**

To investigate the genetic and epigenetic landscape of hypodiploid (<45 chromosomes) acute lymphoblastic leukemia (ALL).

**Methods:**

Single nucleotide polymorphism array, whole exome sequencing, RNA sequencing, and methylation array analyses were performed on eleven hypodiploid ALL cases.

**Results:**

In line with previous studies, mutations in *IKZF3* and *FLT3* were detected in near-haploid (25–30 chromosomes) cases. Low hypodiploidy (31–39 chromosomes) was associated with somatic *TP53* mutations. Notably, mutations of this gene were also found in 3/3 high hypodiploid (40–44 chromosomes) cases, suggesting that the mutational patterns are similar in low hypodiploid and high hypodiploid ALL. The high hypodiploid ALLs frequently displayed substantial cell-to-cell variability in chromosomal content, indicative of chromosomal instability; a rare phenomenon in ALL. Gene expression analysis showed that genes on heterodisomic chromosomes were more highly expressed in hypodiploid cases. Cases clustered according to hypodiploid subtype in the unsupervised methylation analyses, but there was no association between chromosomal copy number and methylation levels. A comparison between samples obtained at diagnosis and relapse showed that the relapse did not arise from the major diagnostic clone in 3/4 cases.

**Conclusion:**

Taken together, our data support the conclusion that near-haploid and low hypodiploid ALL are different with regard to mutational profiles and also suggest that ALL cases with high hypodiploidy may harbor chromosomal instability.

## INTRODUCTION

Hypodiploidy (<46 chromosomes) is found in 5–8% of acute lymphoblastic leukemia (ALL). This karyotypic feature can be further subdivided into a modal chromosome number of 45, high hypodiploidy (HoH; 40–44 chromosomes), low hypodiploidy (HoL; 31–39 chromosomes), and near-haploidy (NH; 25–30 chromosomes) [1–5]. The majority of hypodiploid cases harbor 45 chromosomes, whereas NH, HoL, and HoH are rare, combined comprising less than 1% of BCP ALL [1, 2]. Near-haploid ALL is primarily diagnosed in children and adolescents, and is associated with white blood cell (WBC) counts of < 50 × 10^9^/l and a sex ratio close to 1[1–3]. BCP ALL with HoL, on the other hand, occurs at all ages [2], but in line with NH, it is equally common in males and females and the WBC counts are generally < 50 × 10^9^/l [1, 4]. In childhood ALL, both NH and HoL are associated with a dismal prognosis, with a 3-year event-free survival rate of 30%; adult patients with HoL also have extremely poor overall survival rates of only 20–30% [1, 5–8]. The few studies specifically focusing on the rare HoH group have identified such cases in both childhood and adult BCP ALL [1, 3, 5, 9]. Nachman et al [5] reported that modal chromosome numbers of 40–43 were associated with a poor prognosis in childhood ALL, but their study only included eight cases.

**Figure 1:**
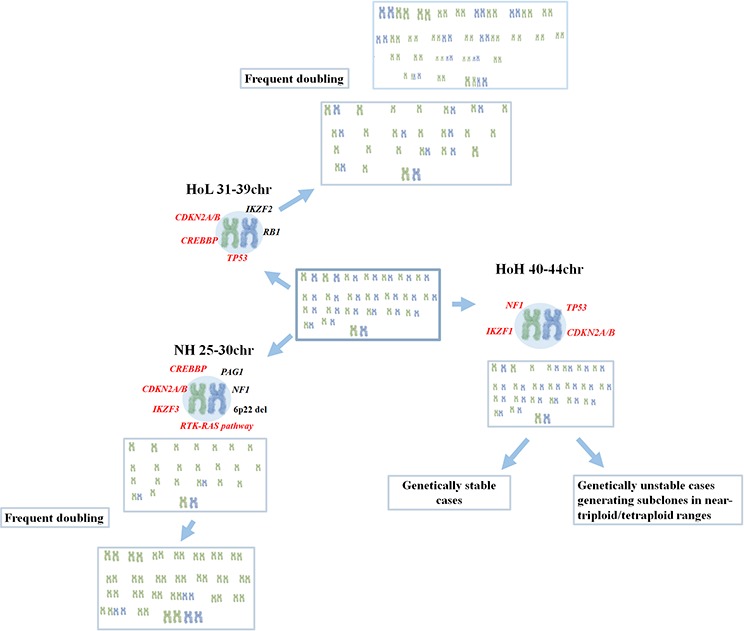
Summary of the genomic landscape of hypodiploid acute lymphoblastic leukemia (ALL) Chromosomal profiles of near-haploid (NH), low hypodiploid (HoL) and high hypodiploid (HoH) ALL are shown, including previously known mutational targets in black and mutational targets identified or confirmed in this investigation in red.

Genetically, NH displays a nonrandom retention of chromosomes X/Y, 14, 18, and 21 [1, 3, 9–12]. A clone constituting a duplication of the stemline is frequently seen and may be mistaken for a high hyperdiploid ALL, a subgroup associated with a favorable outcome [1, 10, 13, 14]. Recurrent microdeletions targeting *CDKN2A/CDNK2B* (22–50%) have been identified in NH ALL [12, 15] and Holmfeldt et al [15] showed that this subtype frequently also harbors mutations and deletions targeting *NF1* (44%), *IKZF3* (13%), and *PAG1* (10%) (Figure 1). The latter study also revealed deletions of the histone gene cluster at 6p22 (19%) and mutations of histone modifier genes. The most common target was *CREBBP* - deletions and sequence mutations of this gene were found in 32% of NH cases. Alterations activating the RTK and RAS signaling pathways, including *NRAS, KRAS, MAPK1, FLT3*, and *PTPN11*, are also frequent (70%) [15] (Figure 1). No recurrent fusion gene has been described.

With regard to HoL ALL, it also retains chromosomes X/Y, 14, 18, and 21. In addition, disomies 1, 5, 6, 8, 10, 11, and 19 are frequent, with disomy 1 being present in close to 100% of cases. In contrast, chromosomes 3, 4, 7, 13, 15, 16, and 17 are monosomic in the majority of cases [1, 4, 12, 16]. In most instances, a second clone with a duplicated chromosome number in the near-triploid range is seen [1, 4]. In fact, because the retained chromosomes in HoL correspond to those gained in near-triploid BCP ALL these two karyotypic groups are often considered to constitute a single entity [7]. Recently, *TP53* mutations were identified in ~90% of pediatric and adult HoL ALL [15, 16] (Figure 1). In pediatric HoL, *TP53* mutations were frequently (43% of cases) detected in nonmalignant hematopoietic cells, strongly suggesting that *TP53* mutations in childhood HoL can be constitutional and that pediatric HoL BCP ALL hence may be a manifestation of Li-Fraumeni syndrome [15]. Adult cases, on the other hand, generally appear to have somatic *TP53* mutations [15, 16]. Also *IKZF2* (53%), *RB1* (41%), and *CDKN2A*/*CDKN2B* (24%) mutations/deletions are prevalent in HoL ALL [15] (Figure 1).

As to HoH, Harrison et al [1] reported that such BCP ALL cases are genetically similar to those with 45 chromosomes, based on the frequent presence of dicentric chromosomes, but their patient cohort comprised only five cases, all of which had 44 chromosomes. In cases with 40–43 chromosomes, available data, albeit scarce, suggest that structural aberrations are common [3, 5]. Doubling of the hypodiploid clone has only been reported in a few cases [3]. To the best of our knowledge, no study has been reported to date where next-generation sequencing has been used to investigate this subgroup.

In the present study, we performed whole exome sequencing (WES), RNA sequencing (RNA seq), and methylation array analyses of NH, HoL, and HoH ALL in order to investigate further the genomic and epigenomic background of hypodiploid ALL.

## RESULTS

### Copy number, microdeletions, and subclonality detected by SNP array analysis

Of the eleven hypodiploid cases included in the study, four were NH, 3 were HoL, and 1 case was HoH. The remaining three cases had G-banding karyotypes showing 79–93 chromosomes. However, data from SNP array analyses showed that many chromosomes harbored complete loss of heterozygosity (LOH), similar to that seen in doubled cases of NH and HoL. We thus deem it likely that they originated via doubling of a hypodiploid clone. However, we cannot determine definitely whether this original clone had a modal number in the HoL or HoH range, since these cases also appear to have chromosomal instability (see below for further information). To account for this uncertainty, we classify these cases as “HoL/HoH” below.

Two HoL cases (#6 and 7) had homozygous loss of part or all of *CREBBP*, which had been missed in the previous analysis of SNP array data. For the HoL/HoH and the HoH case, complete LOH was seen for chromosomes 4, 5, and 16 in 4/4 cases. All HoL/HoH cases (#8, 9 and 10) had allelic ratios indicative of variable copy number, i.e., subclonality, for whole chromosomes. The median number of microdeletions was 1.75 per case in the HoH and HoL/HoH group. Two of the three HoL/HoH cases harbored *CDKN2A/B* deletions; in one of these, the deletion was also seen at relapse (Supplementary Table S1).

### WES results confirms the subtype-specific mutations in hypodiploid ALL

A total of 399 non-synonymous mutations predicted to be deleterious were identified by WES (Supplementary Table S3), of which 24 were in genes that have been previously reported to be mutated in NH and HoL ALL [15]. Recurrently mutated genes included *TP53* (55%), *GPR98* (22%), and *TTN* (22%). The WES analysis also detected mutations in genes known to be mutated in NH ALL, such as *IKZF3* (one relapse sample) and *FLT3* (1/3 cases). In HoL, *TP53* alterations were found in both cases analyzed (100%); the MAFs of these mutations suggested that they were acquired rather than constitutional. In the three HoL/HoH cases analyzed, *NFI* mutations were identified in matching diagnostic and relapse material in one case, *IKZF1* alterations in one case (also at relapse), and *TP53* mutations in all three cases, including the relapse sample in case 9 (Supplementary Table S3).

### Clonal relationship between diagnosis and relapse samples

In all three cases for which paired diagnostic and relapse samples were available, the relapse samples harbored more mutations (Table 2). The clonal relationship between paired diagnostic and relapse samples could be successfully compared by SNP array analysis in cases 2, 4, 5, and 9 and by WES in cases 2, 4, and 9 (Table 2). Cases 5 and 9 displayed differences in copy number alterations between diagnosis and relapse, whereas cases 2, 4, and 9 displayed different mutations in both diagnostic and relapse samples (Table 2).

Mutated genes unique for a diagnostic or relapse sample included *PARD3B* at diagnosis and *TAOK2* and *CREBBP* at relapse in case 2. In case 4, *FLT3* and *PAX9* mutations were seen at relapse only and in case 9, *PIK3C2B* was mutated only at relapse.

### Geneset enrichment analysis reveals higher expression of genes on heterodisomic chromosomes in NH and HoL ALL

The DeSeq gene expression analysis revealed two genes to be upregulated in hypodiploid cases compared with the controls, namely *IGFBP2* and *PARD3B*. Unsupervised principal component analysis (PCA) of NH, HoL, and control cases identified that they clustered separately (Supplementary Figure S1a). Differential expression analysis (variance 0.68) between NH, HoL, and control cases resulted in 76 differentially expressed probe sets. Supervised hierarchical clustering analysis on NH, HoL, and control (FDR < 0.01) revealed 160 differentially expressed genes (Supplementary Figure S1b). GSEA showed possible enrichment of genes on chromosomes X, Y, 14, and 21 in NH cases and of genes on chromosomes X, Y, 1, 5, 6, 8, 10, 11, 18, 19, 21, and 22 in HoL cases, but none of the expression differences was statistically significant. GSEA results from Holmfeldt et al [15] for HoL cases, showed enrichment of genes on chromosomes 1 (*P*-value: 0.004, *q*-value: 0.174), 8 (*P*-value: 0.009, *q*-value:0.146), 10 (*P*-value: 0.004, *q*-value: 0.251), 11 (*P*-value: 0.032, *q*-value: 0.128), 21 (*P*-value: 0.017, *q*-value: 0.149), and X (*P*-value: 0.021, *q*-value:0.214). For the NH cases the analysis showed enrichment of genes on chromosomes 21 (*P*-value: 0.009, *q*-value: 0.027) and X (*P*-value: 0.005, *q*-value: 0.034). No fusion transcripts could be confirmed in the three samples analyzed.

### Methylation status confirms the divergent profiles in NH, HoL, and HoL/HoH ALL

Unsupervised PCA of methylation array data from all hypodiploid cases, including matching relapse samples (*n* = 12), revealed that NH, HoL, and HoL/HoH cases formed distinct clusters (variance = 0.75). When restricting the analysis to commonly lost or retained chromosomes, similar clusters were formed in an unsupervised PCA (Supplementary Figure S2). To investigate whether the methylation status of genes differed between NH, HoL, HoL/HoH, and control samples, we analyzed methylation array results in a supervised multigroup PCA. The analysis revealed 143 genes with significantly different methylation patterns in the NH, HoL, HoL/HoH, and control groups (*P* < 0.05, FDR < 0.1) (Supplementary Figure S3). We performed an unsupervised multigroup analysis (variance 0.2) comparing the mean β values of all NH and HoL diagnostic cases and controls, including an additional analysis between NH and HoL/HoH at diagnosis and relapse. The analysis did not detect any significant methylation level changes between hypodiploid samples and controls. Furthermore, there were no statistically significant changes in methylation levels between diagnostic and relapse samples.

### FISH analysis identifies clonal heterogeneity in HoL/HoH ALL

For the HoL/HoH cases, the SNP array analysis results suggested subclonality of whole chromosomes. Interphase and metaphase FISH was performed to investigate chromosomal copy numbers for these cases (Supplementary Table S4), including all chromosomes in cases 8 and 10 and a subset of chromosomes in case 9. Variable copy numbers were detected for the majority of chromosomes, including both chromosomes that showed retained heterozygosity and those that showed complete LOH (Supplementary Table S4).

## DISCUSSION

Cytogenetically, three genetic subtypes of hypodiploid ALL have been delineated, namely NH, HoL, and HoH. Recently Holmfeldt et al [15] showed that NH and HoL cases have different mutational profiles, indicating that this classification identifies true biological differences in childhood cases. In the present study, we confirm several of the subtype-specific mutations in additional hypodiploid cases (Figure 1), investigate adult cases, and add new insight into the pathogenesis of HoL/HoH ALL.

The chromosomal patterns seen in NH and HoL cases agreed well with what has been previously published [1, 3, 9, 15]. In line with Holmfeldt et al [15], *IKZF3* and *FLT3* mutations were detected in NH cases, 2/2 HoL cases investigated by WES had *TP53* mutations, and 2/3 HoL cases analyzed by SNP arrays had *CDKN2A* deletions. However, whereas Holmfeldt et al [15] found that alterations affecting *CREBBP* were restricted to NH, 2/3 HoL cases in our study had microdeletions of part of this gene. Since their study included only pediatric cases and all our HoL patients were adults, it is possible that *CREBBP* deletions may be pathogentically important primarily in adult HoL ALL.

The present study included 1 case of HoH ALL, a very rare subgroup that has been little investigated, and three additional possible HoH cases. The latter were classified as hypodiploid ALL based on the results from SNP array analysis, which showed LOH for a subset of the chromosomes (Table 1), similar to the pattern seen in NH and HoL cases with doubling of chromosomes. Notably, all three cases with widespread LOH harbored substantial heterogeneity in chromosomal numbers between cells, indicative of chromosomal instability (CIN) (Supplementary Table S4). The HoH case with 40 chromosomes did not have a doubled clone and did not show any evidence of CIN, although many microdeletions were detectable by SNP array analysis (Supplementary Table S1). We have found SNP array data from two additional HoH cases, both with 43 chromosomes, in the literature [15]; none of these harbored evidence of clonal heterogeneity in chromosomal numbers. Thus, some cases with modal numbers in the upper HoL and HoH range may be characterized by chromosomal doubling followed by CIN. As regards specific gene targets, *CDKN2A* deletions have been identified in the two HoH cases investigated by SNP array analysis [15] and in two out of three HoL/HoH cases investigated in our study. To the best of our knowledge, no previous study has used WES to investigate HoH ALL, but targeted gene sequencing has been reported for three cases; all of those had *TP53* mutation [15, 16]. In line with this, all our three HoL/HoH also had such mutations. Based on this, we suggest that HoH BCP ALL may be genetically similar to HoL ALL and should perhaps be classified as such.

**Table 1:** Basic clinical and genetic features of the 11 hypodiploid BCP ALL cases

Case No.	Sex/age	Genetic subtype	Karyotype	Whole chromosome changes detected by SNP array analysis*	Analyses performed			
					WES	RNA seq	Meth array	FISH
1Dx	F/1	NH	25, X, +X, +21/50, idemx2	25, X, +X, +21	Yes	No	Yes	No
2Dx	F/2	NH	Failure	27, X, +X, +14, +18, +21	Yes	No	Yes	No
2R	F/4		Failure	27, X, +X, +14, +18, +21	Yes	No	Yes	No
3R	M/4	NH	29, X, +X, +Y, +Y, +14, +18, +21/58, idemx2	29, X, +X, +Y, +Y, +14, +18, +21	Yes	No	Yes	No
4Dx	F/12	NH	51–52, XX, +X, +21, inc	26, X, +X, +14, +21	Yes	Yes	Yes	No
4R	F/15		Normal	26, X, +X, +14, +21	Yes	Yes	Yes	No
5Dx	M/48	HoL	Normal	32, X, +1, +5, +6, +8, +10, +14, +19, +21, +22	Yes	No	Yes	Yes
5R	M/49		Normal	32, X, +1, +5, +6, +8, +10, +14, +19, +21, +22	No	Yes	No	Yes
6Dx	F/56	HoL	Normal	34, X, +X, +1, +2, +6, +10, +11, +12, +14, +18, +21, +22	Yes	No	Yes	No
7Dx	M/62	HoL	33, X, +Y, +1, +6, +10, +11, +14, +18, +19, +21, +22, inc/61–65, idemx2, inc	33, X, +Y, +1, +5, +6, +10, +11, +18, +19, +21, +22	No	Yes	No	No
8Dx	F/32	HoL/HoH	84, XXXX, −2, −3, −4, −5, +6, −7, −8, −9, +10, +14, −15, −15, −16, −17, −17, +18, −19, +21	Complete LOH for chr3, chr4, chr5, chr7, chr9, chr13, chr15, chr16, chr17, chr20	Yes	No	Yes	Yes
9Dx	M/52	HoL/HoH	Failure	Varying copy number for all chromosomes	Yes	No	Yes	No
9R	M/52		84–93, XX, +X, −Y, −Y, +add(1)(p36), −3, −4, +6, +6, +?der(6;15)(p10;q10), −7, der(11) t(11;11)(p11;q13), −14, −15, −15, −15, −16, −18or +18??,+21, +21, −22, +6mar	Varying copy number for all chromosomes	Yes	No	Yes	Yes
10Dx	F/70	HoL/HoH	79–80, XXX, +1, +2, −4, −8, −9, +11, +12, −16, inc	Complete LOH for chr3, chr4, chr7, chr8, chr9, chr13, chr14, chr15, chr16, chr17, chr20, chr22	Yes	No	Yes	Yes
11Dx	F/75	HoH	40, XX, add(1)(q31), −3, −4, −5, add(7)(q11), −9, −11, −13, −16, −17, −17, −18, +4mar	Complete LOH for chr4, chr5, chr16	No	No	No	Yes

BCP ALL - B-cell precursor acute lymphoblastic leukemia, Dx - diagnosis, F - female, FISH - fluorescence in situ hybridization, HoH - high hypodiploid (40–44 chromosomes), HoL - low hypodiploid (31–39 chromosomes), LOH - loss of heterozygosity, M – male, Meth - methylation, NH - near-haploid (25–30 chromosomes), R - relapse, SNP - single nucleotide polymorphism, WES - whole exome sequencing.

* Copy number changes are given in relation to the haploid level except for cases 8–11. All chromosomes that were not gained displayed complete loss of heterozygosity in cases 1–7.

**Table 2:** Clonal relationship between diagnosis and relapse in hypodiploid ALL

Case No.	Subgroup	Genomic imbalances seen only at diagnosis or relapse Position (bp)	Unique mutations detected only at dx/relapse	Genetic relationship
2D 2R	NH	None None	8 84	Ancestral clone
4D 4R	NH	None None	8 317	Ancestral clone
5D 5R	HoL	None del(chr1:210, 984, 565-qter)	–	Major clone
9D 9R	HoL/HoH	Subclonal+1, subclonal +12, subclonal +21 Subclonal del(chr12:76, 900, 956–91, 479, 154)	1 12	Ancestral clone

bp - base pair, chr - chromosome, D - diagnostic sample, del - deletion, dup - duplication, HoH - high hypodiploidy, HoL- low hypodiploidy, NH - near-haploidy, qter - q-terminal, R - relapse sample.

The genomic landscape of hypodiploid ALL is characterized by aneuploidy and mutations, with few microdeletions and structural rearrangements. This is similar to high hyperdiploid (51–67 chromosomes) childhood ALL [26], which is associated with massive chromosomal gains instead of losses. Thus, the pathogenetic mechanisms may be similar in these two disorders, although the clinical outcomes differ substantially. In both ALL subtypes, aneuploidy seems to be the main driver event, but its functional consequences are still poorly understood, although dosage effects are likely to be important. The fact that different tumors displaying massive hypodiploidy retain different chromosomes in a heterodisomic state supports this scenario [27].

In inflammatory leiomyosarcoma with NH, it has been shown that the loss of chromosomes leads to a general decrease in expression from genes on these chromosomes.^28^ In the present study, RNA-seq data were only available from 3 cases, hampering analyses of gene expression on a global scale. Therefore, we also performed a separate GSEA using data from Holmfeldt et al [15]. These analyses showed that genes on the most commonly retained chromosomes in NH and HoL cases were enriched in these subtypes compared with near-diploid/diploid ALL, indicating that the aneuploidy is associated with specific gene dosage effects. Another possibility is that the monosomic chromosomes display a different methylation pattern, which could also affect gene expression. However, we could not detect any general differences in methylation between chromosomes present in one or two copies in NH and HoL. In line with this, unsupervised methylation analysis resulted in clusters for NH, HoL, and HoL/HoH that remained when the analyses were restricted to genes on the retained chromosomes or genes on the lost chromosomes, indicating the differences in methylation levels were not due to chromosomal copy number (Supplementary figure S2). It should be noted that the methylation arrays used in the present investigation primarily includes CpGs in promotors, and it is hence possible the monosomic chromosomes exhibit different methylation patterns outside of these regions. Paired diagnostic and relapse samples could be successfully investigated for four cases – two NH, one HoL, and one HoL/HoH (Table 2). These analyses showed copy number changes and/or mutations that were unique for the diagnostic or relapse sample, respectively, in cases 2, 4, and 9, indicating that the relapse did not arise from the major diagnostic clone but rather from an ancestral clone. In case 5, unique copy number changes were seen only in the relapse sample, compatible with a scenario where the relapse developed from the major diagnostic clone; however, since no mutation analysis was done in this case, the clonal relationship cannot be definitively determined. Notably, all relapse samples showed many more mutations than the corresponding diagnostic sample (Table 2), indicating rapid selection and possibly an increased mutation rate, both of which could be treatment-related.

Taken together, our results confirm the mutational differences recently described between NH and HoL ALL, and suggest that adult HoL is genetically similar to childhood cases. It is notable that the current WHO classification groups all hypodiploid ALL together (WHO classification of tumours of haematopoietic and lymphoid tissues)[28]. The data from Holmfeldt et al [15], Mülbacher et al [16], and this study suggest that at least NH and HoL should be considered separate entities, with the latter also being defined by *TP53* mutations in addition to the hypodiploidy.

We also provide the first complete genetic characterization of HoH ALL. Although our data are limited, the fact that *TP53* mutations have been found in all HoL/HoH and HoH investigated so far suggests that the pathogenetic mechanism may be similar to the one operating in HoL. However, we also detected evidence for CIN in HoL/HoH; a rare phenomenon in ALL [29]. The doubled clone of HoL ALL is sometimes not an exact duplicate of the hypodiploid clone, indicating that additional copy number changes of whole chromosomes may take place in these cases, but chromosomal gains or losses restricted to a few chromosomes are not necessarily associated with CIN. Thus, to the best of our knowledge, this is the first description of possible CIN in hypodiploid ALL. Further studies will be needed to determine whether CIN is generally present in ALL originating as a clone with modal numbers in the upper HoL/HoH range.

## MATERIALS AND METHODS

### Patients

The study comprised a total of 11 cases with hypodiploid BCP ALL, diagnosed between 1995 and 2012. These included four cases of NH, 3 cases of HoL, 1 case of HoH, and three cases classified as HoL/HoH, as described in the Results section. Of the patients, four were children and seven were adults (range 1–75 years); the cytogenetic and clinical features are summarized in Table 1. All cases were cytogenetically analyzed as part of clinical routine at the Department of Clinical Genetics, University and Regional Laboratories, Region Skåne, Lund, Sweden. SNP array data have been previously published [12, 17, 18]. In total, such data were available for diagnosis and relapse samples from 10 and 5 cases, respectively (Table 1; Supplementary Table S1). Remission material was available for case 3. The investigation was approved by the Research Ethics Committee of Lund University, and informed consent was provided according to the Declaration of Helsinki.

### Whole exome sequencing

WES was performed on nine diagnostic samples, three paired relapse samples, and one additional relapse sample for which diagnostic material was unavailable (Table 1). A remission sample was available only in case 3. Libraries were constructed using the SureSelectXT2 Human All Exon V4 kit (Agilent Technologies, Santa Clara, CA) and cases were subjected to paired-end next generation sequencing (NGS) with an Illumina Hiseq2000 (Illumina, San Diego, CA) by BGI Tech Solutions (Hong Kong). SNP calling was performed using SOAPsnp [19]. For all cases except case 3, the results were further filtered by removing synonymous mutations, neutral mutations (Condel) [20], and variants detected in the 1000 Genomes project (1000g2012apr) and/or the Single Nucleotide Polymorphism Database (dbSNP129). In addition, BAM files were analyzed using the StrandNGS software (Agilent Technologies). Variants in *TP53* were kept if they had been reported as somatic in the COSMIC database (http://cancer.sanger.ac.uk/cancergenome/projects/cosmic/), regardless of the above filtering. In cases 1, 4, 5, and 6, the samples contained a sufficient amount of normal cells to enable a strategy in which where mutations were classified as acquired or constitutional based on their mutant allele fractions (MAFs). In short, if the fraction of leukemic cells in the sample is considered to be x and the fraction of normal cells is 1-x, the MAFs for acquired mutations in monosomic chromosomes is predicted to be x/(x+2(1-x)), the MAFs for constitutional variants in the lost homologue (1-x)/(x+2(1-x)), and the MAFs for constitutional variants in the retained homologue (1x+(1-x)/(x+2(1-x)). Comparisons between diagnostic and relapse samples and between relapse and remission sample in case 3 was done for three cases using MuTect and Indelocator [21].

### RNA sequencing

RNA seq was performed on Illumina Hiseq2000 by BGI Tech Solutions on three cases: one NH, including material from a paired relapse sample, and 2 HoL (Table 1). Five pediatric and adult ALL samples with normal karyotypes were included as controls. Prior to sequencing, the Truseq RNA sample Prep kit (Illumina) was used, for library preparation. Reads were mapped to reference sequences using SOAPaligner/SOAP2 [19], with a read length of 100bp and 100x coverage. To identify candidate fusion transcripts from the sequence data, analyses were performed on FASTQ files applying the ChimeraScan (version 0.4.5), SOAPfuse (version 1.26), and TopHat (version 2.0.7) software. The GRCh37/hg19 build was used as the human reference genome. For gene expression profile analysis, the R package DESeq (version 2.0), using a false discovery rate (FDR) ≤0.01 and a fold change >2, was applied to test for differential gene expression [22]. In addition, statistical analysis and visualization of the RNA sequencing data were performed using Qlucore Omics Explorer 3.1 (Qlucore, Lund, Sweden). Probe sets below a FDR cut-off of 0.01 were considered differentially expressed.

### Methylation array analysis

Methylation array analysis was performed on a total of 9 cases (4 NH, 2 HoL, and 3 HoL/HoH; Table 1) using the HumanMethylation450 Analysis BeadChip and processed according to the Illumina Infinium 450K Methylation array assay procedure (Illumina). The NH cell line Nalm16 and three controls – one pediatric and two adult ALL cases with normal karyotype – were included in the analysis. The data were normalized according to standard procedures using R (version 2.14.0) with Bioconductor packages (version 2.9) [23] and then analyzed using Qlucore Omics Explorer 3.1 (Qlucore). Probe sets below an FDR cut-off of 0.1 were considered differentially methylated. Gene set enrichment analysis (GSEA) [24, 25] was done in Qlucore Omics Explorer 3.1 with gene sets corresponding to whole chromosomes, comparing NH and HoL cases with BCP ALLs with a normal karyotype. Furthermore, GSEA was performed on expression data generated by Holmfeldt et al [15] (GSE27237_RAW; Gene Expression Omnibus database, http://www.ncbi.nlm.nih.gov/geo/), comparing NH cases and controls (*ETV6/RUNX1*-positive ALL) and HoL and controls (*ETV6/RUNX1*-positive ALL).

### Fluorescence in situ hybridization (FISH)

Interphase and metaphase FISH analyses were performed in order to investigate chromosomal copy numbers for cases 8D, 9R, and 10D. FISH was carried out according to standard methods using centromere-specific probes or locus specific probes (Abbott Scandinavia, Stockholm, Sweden). For interphase FISH, a minimum of 200 nuclei were analyzed for each probe.

### RT-PCR

To validate possible fusion genes detected by the RNA seq, RT-PCR was performed according to standard methods. Primers specific for *PIM3, SCO2, N4BP2L1, HMGB1, TPM4, KLF2, ZEB2, CXCR4, B2M*, and *KLC1* were designed to detect possible fusion transcripts (Supplementary Table S2). Products were amplified using an initial denaturation for 2 minutes at 94°C, followed by 30 cycles of 30 seconds at 94°C, 30 seconds at 58°C, and 3 minutes at 72°C, and a final extension for 3 minutes at 72°C.

## SUPPLEMENTARY FIGURES AND TABLES




