# Supplementary material for: Genetic and epigenetic characterization of hypodiploid acute lymphoblastic leukemia
Source: Oncotarget. 2015 Oct 30;6(40):42793–802. doi: 10.18632/oncotarget.6000 (PMC4767471; doi:10.18632/oncotarget.6000)
Supplement: Supplementary file 1 [file oncotarget-06-42793-s001.pdf]

SUPPLEMENTARY FIGURES AND TABLES

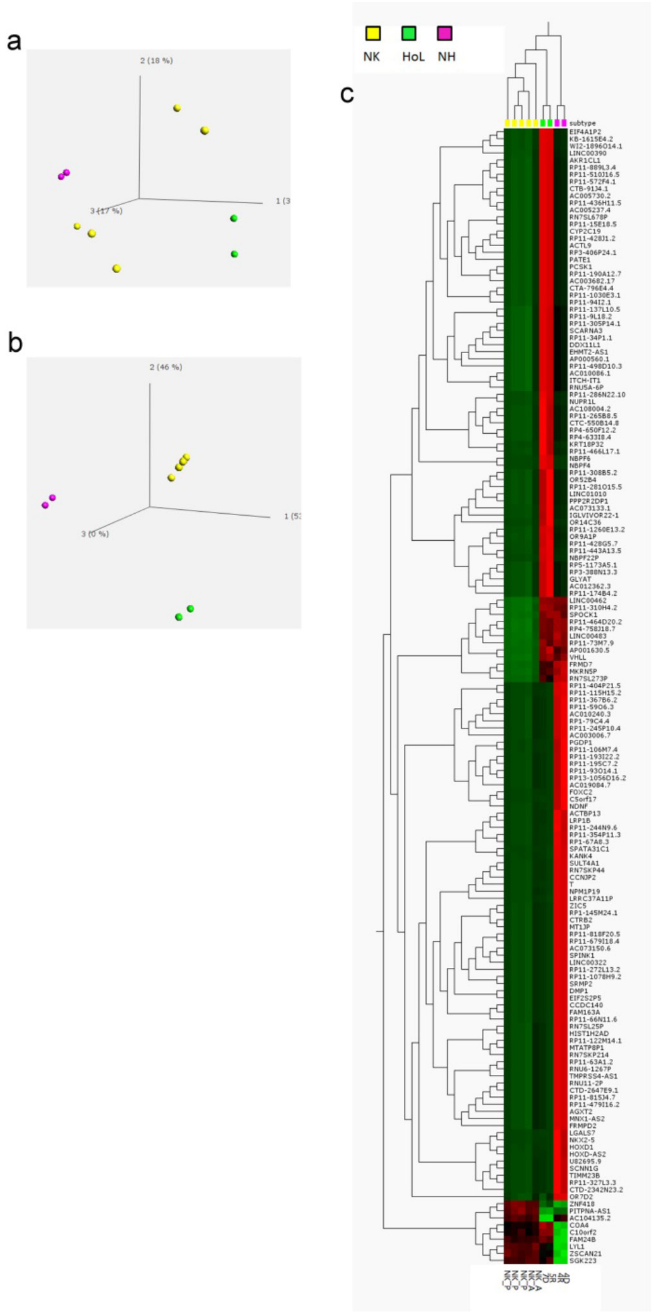

Supplementary Figure S1: A. Unsupervised principal component analysis (PCA) of gene expression data from 4 hypodiploid samples, including five normal karyotype controls. **B.** Supervised PCA of gene expression data from 4 hypodiploid samples, including five normal karyotype controls. C. Hierarchial clustering analysis performed using the 160 most variable probe sets (FDR = 0.01).

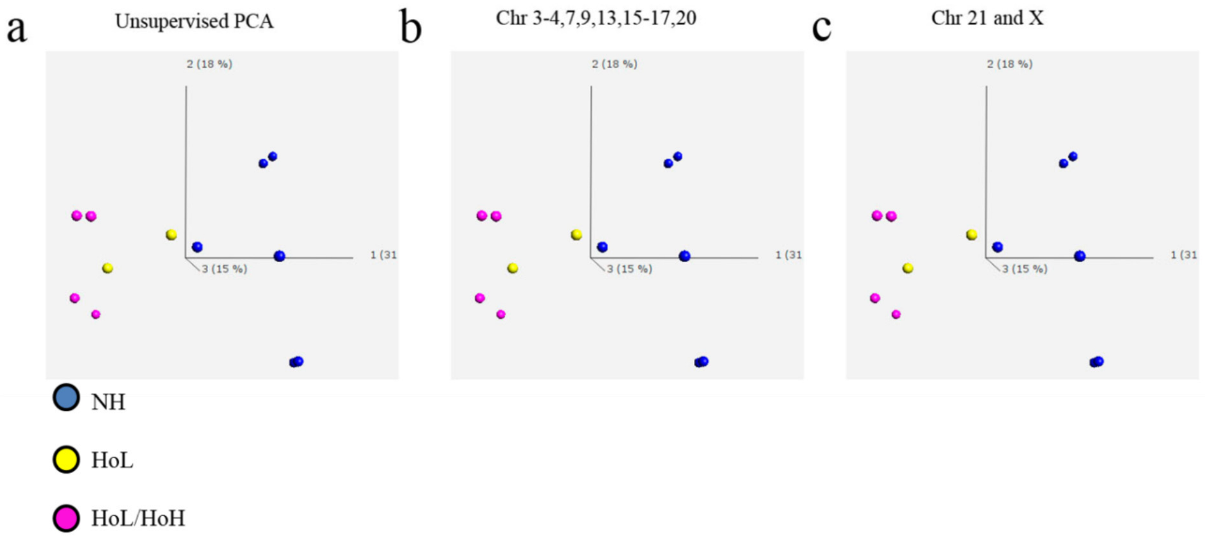

Supplementary Figure S2: A. Unsupervised principal component analysis (PCA) plot of methylation array data from 9 hypodiploid cases. Near-haploid, HoL and HoL/HoH cases form clusters by PCA. **B.** When restricting the analysis to commonly lost chromosomes **C.** or only chromosome 21 and X, retained in both copies in all cases.

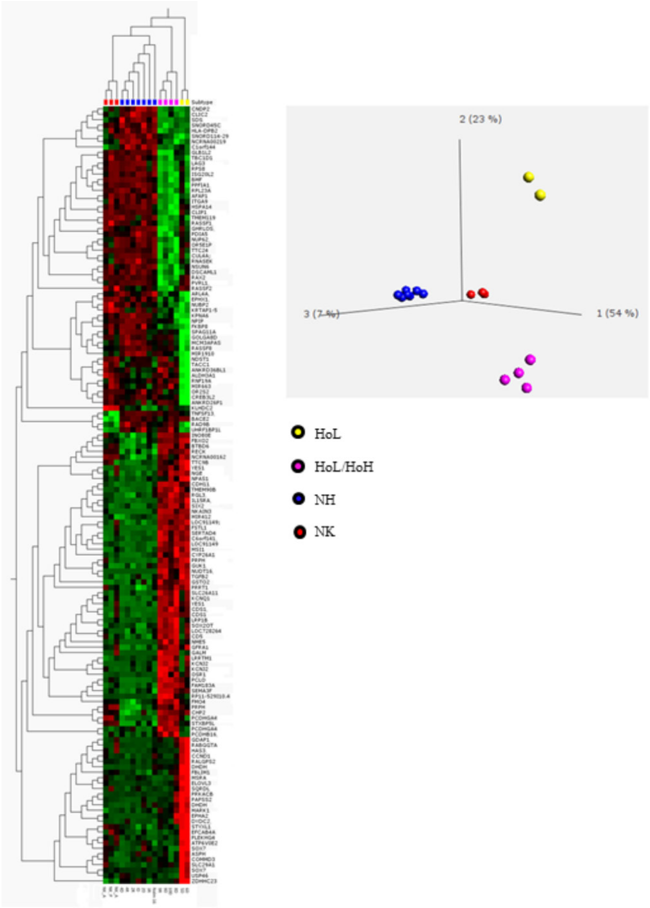

Supplementary Figure S3: A. Hierarchical clustering analysis performed using the 143 most variable probe sets. **B.** Multigroup supervised principal component analysis (PCA) of methylation array data from 9 hypodiploid cases, including a near-haploid cell line, Nalm16 and three normal karyotype controls (FDR = 0.1).

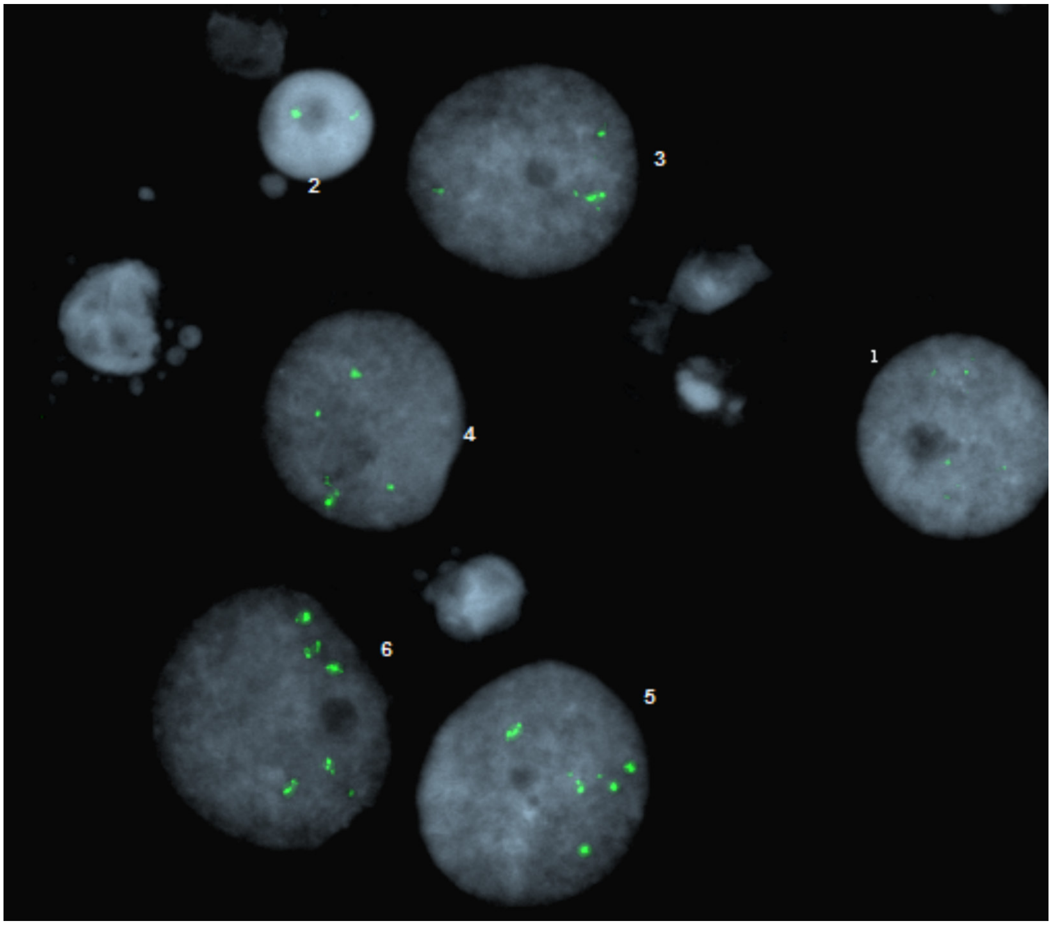

Supplementary Figure S4: Interphase FISH on case 10, showing four or five copies of chromosome 21 in a majority of cells.

Supplementary Table S1: Single nucleotide polymorphism array analysis results for hypodiploid acute lympoblastic leukemia

Supplementary Table S2: Exome sequencing analyses results on 10 hypodiploid cases

Supplementary Table S3: *TP53* mutations in HoL and HoL/HoH ALL

Case No.	Subtype	Gene	Chr	Function	Exonic Biotype	MAF (%)	Cosmic	Condel	Start	End	Ref	Obs
5D	HoL	TP53	17	SPLICE_SITE_ ACCEPTOR		14	COSM984869(1)		7574034	7574034	C	G
6D	HoL	TP53	17	NON_ SYNONYMOUS_ CODING	MISSENSE	69	COSM1636702(5)	0.889, deleterious	7577094	7577094	G	A
8D	HoL/HoH	TP53	17	NON_ SYNONYMOUS_ CODING	MISSENSE	85	COSM99022(89)	0.925, deleterious	7578406	7578406	C	T
9D	HoL/HoH	TP53	17	NON_ SYNONYMOUS_ CODING	MISSENSE	68	COSM99718(31)	0.880, deleterious	7578190	7578190	T	C
9R	HoL/HoH	TP53	17	NON_ SYNONYMOUS_ CODING	MISSENSE	82	COSM99718(31)	0.880, deleterious	7578190	7578190	T	C
10D	HoL/HoH	TP53	17	NON_ SYNONYMOUS_ CODING	MISSENSE	89	COSM1640833(16)	0.840, deleterious	7577548	7577548	C	T

Chr – chromosome, D - diagnostic sample, HoH - high hypodiploid ALL, HoL- low hypodiploid ALL, MAF- mutant allele frequency.

Supplementary Table S4: FISH array analysis results on HoL/HoH ALL

Case no	Chr	1c	2c	3c	4c	5c	6c	SNP BAF
8D	1	0.5%	9.7%	13%	75%	1.5%	–	0.5 (or tetrasomy)
8D	2	–	11%	55%	33%	–	–	Split BAF indicating subclones with more than two copies of chromosome
8D	3	1.3%	18%	80%	–	–	–	Complete LOH
8D	4	2.2%	15%	82%	1.1%	–	–	Complete LOH
8D	5	2.0%	9.0%	89%	–	–	–	Complete LOH
8D	6	–	6.0%	4.0%	17%	73%	–	Split BAF indicating subclones with more than two copies of chromosome
8D	7	1.5%	17%	82%	–	–	–	Complete LOH
8D	8	–	9.0%	11%	79%	1.0%	–	0.5 (or tetrasomy)
8D	9	2.0%	96%	3.0%	2.0%	–	–	Complete LOH
8D	10	–	9.2%	2.4%	29%	59%	–	Split BAF indicating subclones with more than two copies of chromosome
8D	11	1.0%	17%	1.0%	80%	1.0%	–	0.5 (or tetrasomy)
8D	12	0.5%	17%	15%	67%	–	–	0.5 (or tetrasomy)
8D	13	1.3%	21%	78%	–	–	–	Complete LOH
8D	14	–	6.0%	4.0%	31%	59%	–	Split BAF indicating subclones with more than two copies of chromosome
8D	15	12%	87%	–	0.9%	–	–	Complete LOH
8D	16	2.2%	22%	75%	0.5%	–	–	Complete LOH
8D	17	9.6%	88%	2.4%	6.0%	–	-	Complete LOH
8D	18	–	2.0%	4.0%	69%	25%	–	Split BAF indicating subclones with more than two copies of chromosome
8D	19p	7.0%	51%	31%	11%	–	–	0.5 (or tetrasomy)
8D	19q	22%	39%	17%	22%	–	–	0.5 (or tetrasomy)
8D	20	0.6%	15%	78%	6.0%	–	–	Complete LOH
8D	21	–	8.0%	3.0%	38%	51%	–	Split BAF indicating subclones with more than two copies of chromosome
8D	22	1.0%	5.0%	28%	66%	–	–	Split BAF indicating subclones with more than two copies of chromosome
8D	X/X	3.0%	23%	31%	43%	–	–	0.5 (or tetrasomy)
8D	5	14%	67%	7.0%	7.0%	4.0%	–	Complete LOH
9R	17	12%	72%	14%	2.0%	–	–	Complete LOH
9R	18	7.0%	57%	23%	11%	3.0%	–	Split BAF indicating subclones with more than two copies of chromosome
9R	19	20%	66%	7.0%	4.0%	3.0%	–	Split BAF indicating subclones with more than two copies of chromosome

(*Continued*)

Case no	Chr	1c	2c	3c	4c	5c	6c	SNP BAF
10D	1	1.5%	3.6%	7.7%	82%	5.1%	–	0.5 (or tetrasomy)
10D	2	2.2%	12%	5.3%	79%	1.7%	–	0.5 (or tetrasomy)
10D	3	–	23%	75%	2.0%	–	–	Complete LOH
10D	4	–	9.0%	88%	3.0%	–	–	Complete LOH
10D	5	3.0%	33%	63%	1.0%	–	–	Clear Split BAF indicating trisomy
10D	6	–	8.0%	7.0%	49%	36%	1.4%	Split BAF indicating subclones with more than two copies of chromosome
10D	7	8.7%	90%	1.7%	–	–	–	Complete LOH
10D	8	–	1.6%	45%	55%		–	Complete LOH
10D	9	6.3%	93%	–	0.5%	–	–	Complete LOH
10D	10	–	10%	2.8%	32%	55%	–	Split BAF indicating subclones with more than two copies of chromosome
10D	11	–	5.0%	18%	75%	1.0%	1.0%	0.5 (or tetrasomy)
10D	12	0.6%	13%	5.0%	82%	–	–	0.5 (or tetrasomy)
10D	13	6.0%	91%	3.0%	–	–	–	Complete LOH
10D	14	17%	44%	39%	–	–	–	Complete LOH
10D	15	15%	42%	43%	–	–	–	Complete LOH
10D	16	7.4%	90%	2.5%	–	–	–	Complete LOH
10D	17	–	1.3%	6.3%	75%	17%	–	Complete LOH
10D	18	–	4.3%	9.4%	39%	48%	–	Split BAF indicating subclones with more than two copies of chromosome
10D	19	–	5.0%	21%	74%	–	–	0.5 (or tetrasomy)
10D	20	1.0%	15%	83%	1.0%	–	–	Complete LOH
10D	21	–	7.0%	10%	47%	34%	2.0%	Split BAF indicating subclones with more than two copies of chromosome
10D	22	1.0%	18%	78%	3.0%	–	–	Complete LOH
10D	X/Y	1.0%	11%	14%	69%	5.0%	–	0.5 (or tetrasomy)

BAF - B allele frequency, c - copy number, D - diagnostic sample, LOH - loss of heterozygosity, R- relapse sample, SNP - single nucleotide polymorphism.

Supplementary Table S5: Primers used for gene fusion verification

Case	Gene	Primer	Sequence
4D (NH)	*N4BP2L1*	*N4BP2L1*_1Forward	ACATGGCCGGAGAGTCACAA
		*N4BP2L1*_2Forward	ATGGCCGGAGAGTCACAAAAA
	HMGB1	*HMGB1*_1Reverse	TTGGGTGCATTGGGATCCTT
		*HMGB1*_2Reverse	GCAGACATGGTCTTCCACCT
	TPM4	*TPM4*_1Forward	GAGGCGGTGAAACGCAAGAT
		*TPM4*_2Forward	CGGTGAAACGCAAGATCCA
	KLF2	*KLF2*_1Reverse	GAAGGCACGATCGCACAGAT
		*KLF2*_2Reverse	GAGAAGGCACGATCGCACAG
4R	*ZEB2*	*ZEB2*_1Forward	TTTTCTCCCCCACACTTCGC
		*ZEB2*_2Forward	GAGGCGTAACACGTCAGTCC
	CXCR4	*CXCR4*_1Reverse	CCATTGCCCACAATGCCAG
		*CXCR4*_2Reverse	ACGGAAACAGGGTTCCTTCAT
	B2M	*B2M*_1Forward	TGCTCGCGCTACTCTCTCTT
		*B2M*_2Forward	CGAGATGTCTCGCTCCGTG
	KLC1	*KLC1*_1Reverse	TCCTATGCACACAGACGCAG
		*KLC1*_2Reverse	ACGTTATCGCACACAACGTG
7D (HoL)	*PIM3*	*PIM3*_1Forward	CATCGACTTCGGTTCGGGTG
		*PIM3*_2Forward	ACCGCGACATTAAGGACGAA
	SCO2	*SCO2*_1Reverse	GGCTTCTGTTCGCTTTTGCT
		*SCO2*_2Reverse	CTGAGAGAGCCTGTGCCAAG

NH – near-haploid, HoL – low hypodiploid.
